# Scientific Items

**Published:** 1885-03-20

**Authors:** 


### SCIENTIFIC ITEMS.

**System for Steering Balloons and Maintaining a Desired Elevation in the Atmosphere.**—Long before the experiments of Messrs. Renard and Krebbs had been made known (described in *Scientific American*, Vol. LI., No. 13), I devised a system of guiding elliptical balloons by the use of an electric current. This system, which was delivered to the Secretary of the Academy of Sciences of Paris, the 27th of August, 1883, No. 3,697, consists in an elliptical balloon inflated horizontally with gas to a suitable extent to almost balance the weight of the basket, the aeronauts, the motor, and the batteries, but in such manner that the basket should not be raised from the earth by the balloon proper, but should require a certain amount of power to lift it. The form of the balloon is that of a cylinder terminating in points at both extremities, thus offering the best possible resistance to the wind. Underneath, and extending its whole length, the balloon is provided with a sail which keeps it always head to the wind, like the tail of a windmill or weather vane. This sail acts as a sort of pivot in the air, and enables the balloon to be properly guided. The basket is provided with an electric motor connected with suitable batteries.

The motor works a horizontal propeller, which serves to impel the balloon forward and enables it to be moved out of the direction of the wind, if necessary. This propeller is movable on its axis, so that it can attain any desired inclination with reference to the sail. The motor also actuates a vertical stationary propeller situated between the sail and the basket, and which serves to raise the balloon either slowly or rapidly, or simply sustains it at a fixed elevation, according to the desire of the aeronaut. It is seen at once that this variation in the rotation of the propeller changes the ascensional force of the balloon, and sustains it at any desired height. Birds float in the air on the same principle. The basket is provided on its inner side with a semi-spherical parachute, which prevents the too rapid descent of the balloon. The guiding of the car is the most simple feature of the whole apparatus, it being necessary to raise it by mechanical power, on account of its being heavier than the atmosphere.—M. C. SENLECQ, Ardres, France, *in Scientific American.*

**Iridium.**—Iridium is a metal which is likely to have a much more extensive employment than it now enjoys. Hitherto it has been chiefly used in alloy with osmium for tipping gold pens. But an American pen manufacturer has discovered that by fusing the metal at a white heat and adding phosphorus perfect fusion could be obtained, with all the hardness in the resulting material of the iridium itself. For mechanical applications this combination is exceedingly useful, as in the case of pen points; and its adaptability is being proved in many ways. Agate, which has hitherto been employed for fine chemical balances, is now giving place to iridium, which takes a finer edge and is not so liable to catch or break.

Hypodermic needles for surgical use are now made of gold and tipped with the iridium compound, which is not subject to corrosion like the old steel points, and it is also being largely applied to instruments for surveyors and engineers and to electrical apparatus. Iridium can be obtained somewhat abundantly from the Russian plantinum mines in the Ural, and it is found in combination with gold in California. Mr. Dudley, of Cincinnati, is engaged on experiments with the object of plating vessels with iridium, and as the metal resists the action of acids, it is likely that such vessels will be very useful in many chemical operations.—*Chemist and Druggist.*

**What Fossils Teach.**—In a recent lecture Dr. P. H. Carpenter, of Eton College, mentioned the case of Greenland as an illustration of the manner in which the earth's history is read from fossils, those remains of by-gone life which in the middle ages were regarded as "sports of nature." Fossils of four climates, all warmer than the present icy one, are found in that country. Remains of the oak and the maple tell us that the climate was once very similar to that of England to-day, and the coal found lower down, shows that something approaching tropical heat prevailed at an earlier period. The fossil of certain sea creatures appear on the land, and that Greenland once lay beneath the sea and that its water was temperate, while the coral, obtained still lower down, must have grown where the waters were still warmer.—*Ibid.*

**The Electric Light as a Scarf Pin.**—Messrs. Stout, Deadowcroft & Co., whose advertisement appears in another column, are now supplying these curious little electrical devices in first class style. It consists of a minature Edison electrical lamp, attached to a pin, which is fastened in the scarf or neck-tie. A couple of fine wires lead from the lamp to a small battery, made in the form of a book and carried in the pocket. By touching a button, also arranged in one's pocket, the necktie lamp is instantly lighted, and continues as long as the button is pressed. The battery becomes exhausted after considerable use, but may be easily replenished This is a device of genuine excellence, and well illustrates the progress of practical electricity.—*Scientific American.*

**Meteoric Dust.**—A metallic substance in powder or small granules has been sent to the *Science News* laboratory for examination. It proves to be meteoric dust, largely composed of iron, nickel, and silica. Dr. Batchelder, of Pelham, N. H., who sent the specimen, states that he collected the dust on the walk in front of his house after a smart thunder shower. It is probable that large quantities of this material fall upon the earth, but remain unnoticed. Much of the iron found in soils is due to precipitation from interstellar spaces, the particles becoming entangled in our atmosphere.—*Popular Science Monthly.*

